# Prognosticators and Prognostic Nomograms for Leiomyosarcoma Patients With Metastasis

**DOI:** 10.3389/fonc.2022.840962

**Published:** 2022-03-18

**Authors:** YuChi Zou, QianKun Yang, YuTong Wu, HongBo Ai, ZhongXiang Yao, ChengMin Zhang, Fei Luo

**Affiliations:** ^1^ National and Regional United Engineering Lab of Tissue Engineering, Department of Orthopedics, Southwest Hospital, Third Military Medical University (Army Medical University), Chongqing, China; ^2^ Department of Physiology, Third Military Medical University (Army Medical University), Chongqing, China

**Keywords:** leiomyosarcoma, metastasis, prognosticators, nomogram, overall survival, cancer-specific survival

## Abstract

Individual survival prediction and risk stratification are of vital importance to optimize the individualized treatment of metastatic leiomyosarcoma (LMS) patients. This study aimed to identify the prognostic factors for metastatic LMS patients and establish prognostic models for overall survival (OS) and cancer-specific survival (CSS). The data of LMS patients with metastasis between 2010 and 2015 were extracted from the Surveillance, Epidemiology, and End Results (SEER) database. The entire cohort was randomly divided into a training cohort and a validation cohort. The influences of primary tumor site, localized and distant metastases, and sites and number of metastases on the prognosis of metastatic LMS patients were firstly explored by Kaplan–Meier curves and log-rank tests. Furthermore, the effective therapeutic regimens and prognosticators for metastatic LMS patients were also analyzed by Cox analysis. In addition, two prognostic nomograms for OS and CSS were established, and their predictive performances were evaluated by the methods of receiver operating characteristic (ROC) curves, time-dependent ROC curves, calibration curves, and decision curve analysis (DCA). A total of 498 patients were finally collected from the SEER database and were randomly assigned to the training set (N = 332) and validation set (N = 166). No significant differences in OS were observed in patients with distant organ metastasis and localized metastasis. For patients who have already developed distant organ metastasis, the sites and number of metastases seemed to be not closely associated with survival. Patients who received chemotherapy got significantly longer survival than that of their counterparts. In univariate and multivariate Cox analyses, variables of surgery, chemotherapy, age, and tumor size were identified as independent predictors for OS and CSS, and distant metastasis was also independently associated with CSS. The areas under the curve (AUCs) of ROC curves of the nomogram for predicting 1-, 3-, and 5-year OS were 0.770, 0.800, and 0.843, respectively, and those for CSS were 0.777, 0.758, and 0.761, respectively. The AUCs of time-dependent AUCs were all over 0.750. The calibration curves and DCA curves also showed excellent performance of the prognostic nomograms. Metastasis is associated with reduced survival, while the sites and the number of metastases are not significantly associated with survival. The established nomogram is a useful tool that can help to perform survival stratification and to optimize prognosis-based decision-making in clinical practice.

## Introduction

Leiomyosarcoma (LMS) is a rare, malignant, and aggressive type of soft tissue sarcoma, which arises from smooth muscle cells (1). Previous studies have reported that LMS accounts for 5%–10% of all soft tissue sarcomas (2). It can occur anywhere in our body and is classified into different subsets based on the location of the primary tumor, with the commonly involved sites being extremities, retroperitoneum, uterine, bladder, and so on (3–5). Patients with different primary tumor sites have presented various clinicopathological behaviors and overall survival (OS), indicating the fact that the survival of LMS is site-specific. Given this, the current prognostic studies of LMS mainly concentrate on identifying the prognosticators and prognostic tools in one subset (or one subgroup) of LMS patients, such as cutaneous LMS (6), extremity LMS (7, 8), uterine LMS (9), urinary bladder LMS (10), and pulmonary LMS (11).

A series of factors have been identified in previous studies to be correlated to the prognosis of LMS, including stage, tumor grade, tumor size, therapeutic procedures, metastasis, local recurrence, etc., with metastasis being one of the most pivotal prognostic factors for LMS (7, 8, 12, 13). The metastasis in LMS mainly includes two forms, namely, local metastasis and distant metastasis. Various organs may be involved in distant metastasis, such as bone, brain, lung, and liver. In patients with malignancies, not just confined to LMS, those who have developed metastasis usually harvest obviously shorter survival. For initially diagnosed non-metastatic LMS, patients with different primary tumor sites usually showed different survival rates and susceptibility to metastasis (7). However, for patients who have already developed metastasis, whether primary tumor site owns equivalent importance still remains unknown. Furthermore, up to now, most of the prognostic studies mainly focused on one subset of LMS, leading to the scarcity of prognostic information on LMS patients with metastasis as a whole cohort. Besides, some other paramount issues also remain to be elucidated in LMS patients with metastasis. Firstly, it is veiled that whether there existed significant survival differences in patients with distant metastasis and patients with local metastasis. Secondly, in LMS patients with distant metastasis, whether patients with one distant organ metastasis have better prognosis than patients with multiple distant organ metastases. Finally, which therapeutic modality remains to be effective once patients developed metastasis. These problems are of vital importance and can serve as pivotal reference for clinicians to optimize clinical interventions for LMS patients who have developed metastasis. Therefore, it is a necessity to conduct a study in a cohort of LMS patients who suffered metastasis to illuminate these issues.

The nomogram is a widely used statistical and practical tool that can incorporate multiple reliable predictors and provide accurate and individualized prognosis information for clinicians in clinical practice (14, 15). It is widely applied in various malignancies for its user-friendliness and reliable discriminability, including lung cancer (16), bone and soft tissue sarcoma (17), hematogenic tumor (18), and so on. For the purpose of effective risk stratification and reference for personalized therapeutic regimen selection for LMS patients with metastasis, a reliable and effective prognostic prediction model for survival in metastatic LMS patients is needed. However, to the best of our knowledge, prognostic nomograms to predict survival in the cohort of LMS patients with metastasis have not yet been reported in previous studies.

Hence, in this study, we firstly explored the influences of primary tumor site, localized and distant metastases, sites of metastases, and number of metastases on the prognosis of metastatic LMS patients. Secondly, we analyzed the effective therapeutic regimens for LMS patients suffering from metastasis. Finally, we identified the prognosticators for overall survival (OS) and cancer-specific survival (CSS) and constructed prognostic nomograms for OS and CSS based on the identified prognosticators, and we also evaluated the predictive performance of the established nomograms.

## Materials and Methods

### Study Population

**Figure 1:**
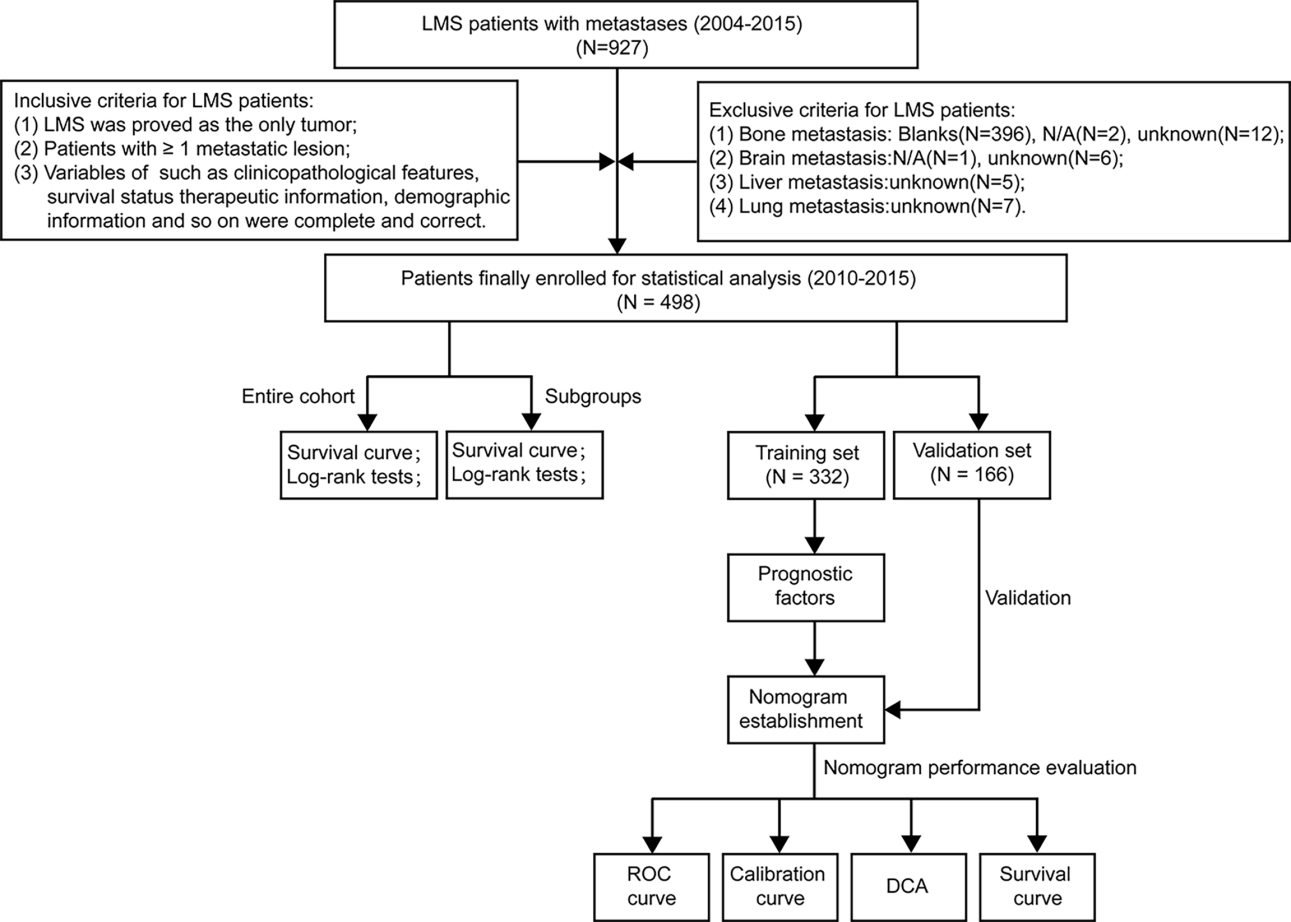
The flowchart describing the process of conducting the study and statistical analysis. ROC, receiver operating characteristic; DCA, decision curve analysis; LMS, leiomyosarcoma.

The workflow of our study design and statistical analyses was illustrated in **Figure 1**. The data of our study cohort were extracted from the Surveillance, Epidemiology, and End Results (SEER) Database. The suggested citation for the selected database was SEER Program (www.seer.cancer.gov) SEER*Stat Database: Incidence–SEER Research Plus Data, 9 Registries, Nov 2020 Sub (1975-2018)–Linked To County Attributes–Total U.S., 1969–2019 Counties, National Cancer Institute, DCCPS, Surveillance Research Program, released April 2021, based on the November 2020 submission. The variables were collected with the software of SEER*Stat (version 8.3.9.2, released on August 20, 2021).

Patients who meet the following criteria were included in this study: (I) pathologically diagnosed as LMS [International Classification of Diseases (ICD)-O-3/WHO 2008 histology codes for LMS were 8890, 8891, and 8896]; (II) LMS was proven as the only primary tumor; (III) got M1 in the derived American Joint Committee on Cancer (AJCC) staging system or with at least one metastatic lesion; (IV) with detailed and complete information on the variables of sex, year of diagnosis, race, marital status, age, tumor size, primary site, SEER cause-specific death classification, SEER other cause of death classification, vital status, chemotherapy, radiotherapy, surgery, collaborative stage (CS) tumor size, and so on. Conversely, patients with unknown or incomplete information on the variables of bone metastasis, brain metastasis, lung metastasis, and liver metastasis were excluded from this study.

### Ethics Statement

The datasets to perform our study all came from the publicly available SEER database (https://seer.cancer.gov/). All information for patients has been specially anonymized before uploading to the database, and thus personally identifiable information was not included in the SEER database. Therefore, the informed consent and ethics approval for this study were waived by the ethics committee of our hospital.

### Variable Encoding and Interpretation

In this study, the following variables were collected from the SEER database, including sex (male, female), age (<58, 58–76, >76), tumor size (≤16.0 cm, >16.0 cm), year of diagnosis (2010–2012, 2013–2015), race (white, black, others), marital status (married, not married, others), regional lymph node surgery (no, yes), surgery (no, yes), radiotherapy (no, yes), chemotherapy (no, yes), systemic therapy (no, yes), AJCC T (T0, T1, T2, Tx), AJCC N (N0, N1, N2), primary site (extremity and trunk, retroperitoneum, others), brain metastasis (no, yes), bone metastasis (no, yes), liver metastasis (no, yes), lung metastasis (no, yes), lung metastasis only (no, yes), bone metastasis only (no, yes), liver metastasis only (no, yes), sum of metastases (blank, 1 metastatic sites, 2 metastatic sites, 3 metastatic sites, 4 metastatic sites), sum of metastases (≤2 metastatic sites, >2 metastatic sites), and distant metastasis (no, yes). During the process of data collection, the variables of AJCC M (M1) and ICD-O-3/WHO 2008 histology (ICD-O-3: 8890, 8891, 8896) were used to ensure that all LMS patients got metastasis. Subsequently, the variables of brain metastasis, bone metastasis, liver metastasis, lung metastasis, and distant metastasis were utilized to identify whether the patients got a localized metastasis or distant metastasis (mainly including brain, bone, liver, and lung). Two continuous variables, including age and tumor size, were changed into categorical variables with the optimal cutoff values identified by the X-tile software. In this study, the main endpoints were OS and CSS. OS was defined as the time interval from disease diagnosis to death due to any kind of cause. CSS was defined as the time interval from disease diagnosis to death merely due to LMS.

### Statistical Analysis

All statistical analyses were performed by IBM SPSS Statistics 26, EmpowerStats (version 2.2) and R software (version 4.1.1). The entire dataset was randomly divided into a training set (N = 332) and validation set (N = 166). The comparisons of demographic and clinicopathological characteristics between the training set and validation set were conducted by Fisher’s exact test or chi-square test for categorical variables. In the whole cohort and in the subgroup of distant organ metastasis, Kaplan–Meier method and log-rank test were utilized to compare the differences in OS and CSS of prognostic predictors. On the basis of metastatic sites, the entire cohort was divided into seven subgroups; the relationship among metastatic sites, treatment modalities, and survival was analyzed by the methods of Kaplan–Meier curves, log-rank test, and Cox regression analysis. A two-tailed P-value <0.05 was regarded as statistically significant.

In the training set, univariate Cox regression analysis was utilized to identify possible prognostic factors for OS and CSS, and factors whose P-values were lower than 0.05 were included in the multivariate Cox analysis. In multivariate Cox analysis, factors with a P-value <0.05 were deemed independent prognostic factors for OS and CSS, and these prognostic predictors were further used for nomogram construction. According to the identified independent prognostic factors, nomograms to predict the 1-, 3-, and 5-year OS and CSS were established with R software. For the evaluation of nomogram performance, the methods of receiver operating characteristic (ROC) curves, calibration curves, time-dependent areas under the curve (AUCs), as well as the C-indexes were utilized to evaluate the discriminability and accuracy of the established nomograms. Furthermore, decision curve analysis (DCA) was used to evaluate the net benefits of the nomogram for LMS patients with metastasis. Finally, based on the median of risk scores of all LMS patients calculated from the established nomograms, the whole cohort was divided into two subgroups, namely, the low-risk group and the high-risk group. The Kaplan–Meier curves and log-rank test were performed to compare the survival differences between the two subgroups to further validate the prognostic value of the established nomograms.

## Results

### Baseline Demographic and Clinicopathological Characteristics of Leiomyosarcoma Patients

**Figure 2:**
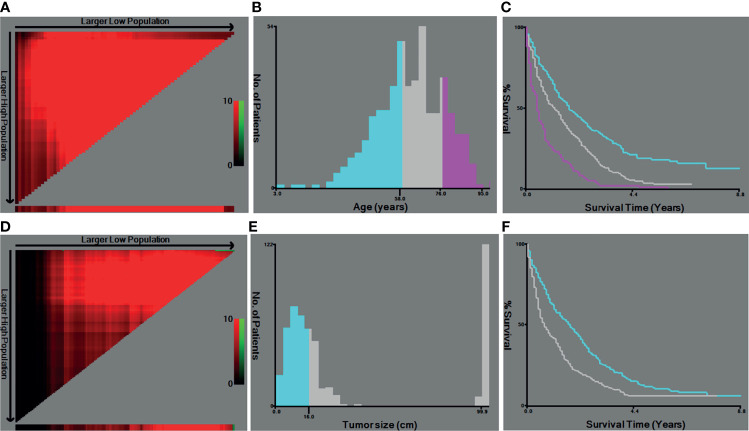
The optimal cutoff values of age and tumor size identified by X-tiles software. **(A, B)** The optimal cutoff value of age. **(C)** The Kaplan–Meier curves for the subgroups of age (<57, 58–75, >76) for OS. **(D, E)** The cutoff value of tumor size. **(F)** The Kaplan–Meier curves for the subgroups of tumor size (<1.6 cm, ≥1.6 cm) for ROC, receiver operating curve; OS. OS, overall survival.

Based on the inclusive and exclusive criteria, a total of 498 LMS patients with metastasis were finally enrolled for statistical analysis. The entire cohort was randomly divided into a training cohort (N = 332) and validation cohort (N = 166). The baseline demographic and clinicopathological characteristics of patients in the whole cohort, training cohort, and validation cohort were presented in **Table 1**. Regarding the variables of age and tumor size, they were changed into categorical variables based on the optimal cutoff values identified by X-tile software **(** **Figure 2** **)**. No significant statistical differences were found in demographic and clinicopathological characteristics of patients in the training set and validation set (all P-value >0.05).

**Table 1:** Baseline demographic and clinicopathological characteristics of LMS patients with metastasis in the whole dataset, training dataset, and validation dataset.

Variables	Total set	Training set	Validation set	P-value
	(N = 498), n (%)	(N = 332), n (%)	(N = 166), n (%)	
Sex				0.949
Male	203 (40.76%)	135 (40.66%)	68 (40.96%)	
Female	295 (59.24%)	197 (59.34%)	98 (59.04%)	
Year of diagnosis				0.800
2010–2012	245 (49.20%)	162 (48.80%)	83 (50.00%)	
2013–2015	253 (50.80%)	170 (51.20%)	83 (50.00%)	
Race				0.494
White	371 (74.50%)	248 (74.70%)	123 (74.10%)	
Black	89 (17.87%)	56 (16.87%)	33 (19.88%)	
Others	38 (7.63%)	28 (8.43%)	10 (6.02%)	
Marital status				0.933
Married	260 (52.21%)	175 (52.71%)	85 (51.20%)	
Not married	99 (19.88%)	66 (19.88%)	33 (19.88%)	
Others	139 (27.91%)	91 (27.41%)	48 (28.92%)	
Regional lymph node surgery				0.910
No	455 (91.37%)	303 (91.27%)	152 (91.57%)	
Yes	43 (8.63%)	29 (8.73%)	14 (8.43%)	
Surgery				0.321
No	321 (64.46%)	209 (62.95%)	112 (67.47%)	
Yes	177 (35.54%)	123 (37.05%)	54 (32.53%)	
Radiotherapy				0.209
No	377 (75.70%)	257 (77.41%)	120 (72.29%)	
Yes	121 (24.30%)	75 (22.59%)	46 (27.71%)	
Chemotherapy				0.073
No	212 (42.57%)	132 (39.76%)	80 (48.19%)	
Yes	286 (57.43%)	200 (60.24%)	86 (51.81%)	
Systemic therapy				0.769
No	374 (75.10%)	248 (74.70%)	126 (75.90%)	
Yes	124 (24.90%)	84 (25.30%)	40 (24.10%)	
AJCC T				0.636
T0	5 (1.00%)	2 (0.60%)	3 (1.81%)	
T1	41 (8.23%)	28 (8.43%)	13 (7.83%)	
T2	292 (58.63%)	194 (58.43%)	98 (59.04%)	
Tx	160 (32.13%)	108 (32.53%)	52 (31.33%)	
AJCC N				0.294
N0	348 (69.88%)	238 (71.69%)	110 (66.27%)	
N1	77 (15.46%)	51 (15.36%)	26 (15.66%)	
N2	73 (14.66%)	43 (12.95%)	30 (18.07%)	
Primary site				0.937
Extremity+trunk	238 (47.79%)	157 (47.29%)	81 (48.80%)	
Retroperitoneum+Peritoneum	109 (21.89%)	74 (22.29%)	35 (21.08%)	
Others	151 (30.32%)	101 (30.42%)	50 (30.12%)	
Age				0.086
<58	198 (39.76%)	127 (38.25%)	71 (42.77%)	
58–76	201 (40.36%)	145 (43.67%)	56 (33.73%)	
>76	99 (19.88%)	60 (18.07%)	39 (23.49%)	
Tumor size				0.611
≤16.0 cm	266 (53.41%)	180 (54.22%)	86 (51.81%)	
>16.0 cm	232 (46.59%)	152 (45.78%)	80 (48.19%)	

LMS, leiomyosarcoma; AJCC, American Joint Committee on Cancer.

In this study, all LMS patients suffered metastasis, but the number of metastatic sites and the location of metastasis varied in different patients. The baseline characteristics of metastasis in LMS patients in the whole cohort, training set, and validation set were shown in **Table 2**. Among the 498 patients, 384 (77.11%) patients suffered distant organ metastasis, and 114 (22.89%) patients got localized metastasis. The main distant metastatic sites for LMS patients were brain, bone, liver, and lung. Here, 154 (30.92%) LMS patients got at least two metastatic sites, while 275 (55.22%) LMS patients got one metastatic site. There existed no significant statistical differences in the variables of metastasis between the training set and validation set (all P-value >0.05).

**Table 2:** Baseline characteristics of metastasis in LMS patients in the whole dataset, training set, and validation set.

Variables	Total set	Training set	Validation set	P-value
	(N = 498), n (%)	(N = 332), n (%)	(N = 166), n (%)	
Brain metastasis				0.369
No	482 (96.79%)	323 (97.29%)	159 (95.78%)	
Yes	16 (3.21%)	9 (2.71%)	7 (4.22%)	
Bone metastasis				0.764
No	382 (76.71%)	256 (77.11%)	126 (75.90%)	
Yes	116 (23.29%)	76 (22.89%)	40 (24.10%)	
Liver metastasis				0.522
No	284 (57.03%)	186 (56.02%)	98 (59.04%)	
Yes	214 (42.97%)	146 (43.98%)	68 (40.96%)	
Lung metastasis				0.371
No	217 (43.57%)	140 (42.17%)	77 (46.39%)	
Yes	281 (56.43%)	192 (57.83%)	89 (53.61%)	
Lung metastasis only				0.673
No	357 (71.69%)	236 (71.08%)	121 (72.89%)	
Yes	141 (28.31%)	96 (28.92%)	45 (27.11%)	
Bone metastasis only				0.463
No	462 (92.77%)	306 (92.17%)	156 (93.98%)	
Yes	36 (7.23%)	26 (7.83%)	10 (6.02%)	
Liver metastasis only				0.577
No	400 (80.32%)	269 (81.02%)	131 (78.92%)	
Yes	98 (19.68%)	63 (18.98%)	35 (21.08%)	
Brain+Bone				0.614
No	490 (98.39%)	326 (98.19%)	164 (98.80%)	
Yes	8 (1.61%)	6 (1.81%)	2 (1.20%)	
Brain+Liver				0.59
No	491 (98.59%)	328 (98.80%)	163 (98.19%)	
Yes	7 (1.41%)	4 (1.20%)	3 (1.81%)	
Brain+Lung				0.443
No	484 (97.19%)	324 (97.59%)	160 (96.39%)	
Yes	14 (2.81%)	8 (2.41%)	6 (3.61%)	
Bone+Liver				0.827
No	452 (90.76%)	302 (90.96%)	150 (90.36%)	
Yes	46 (9.24%)	30 (9.04%)	16 (9.64%)	
Bone+Lung				0.4
No	432 (86.75%)	291 (87.65%)	141 (84.94%)	
Yes	66 (13.25%)	41 (12.35%)	25 (15.06%)	
Liver+Lung				0.185
No	394 (79.12%)	257 (77.41%)	137 (82.53%)	
Yes	104 (20.88%)	75 (22.59%)	29 (17.47%)	
Brain+Bone+Liver				0.219
No	495 (99.40%)	329 (99.10%)	166 (100.00%)	
Yes	3 (0.60%)	3 (0.90%)	0 (0.00%)	
Brain+Bone+Lung				0.384
No	492 (98.80%)	327 (98.49%)	165 (99.40%)	
Yes	6 (1.20%)	5 (1.51%)	1 (0.60%)	
Bone+Liver+Lung				0.802
No	464 (93.17%)	310 (93.37%)	154 (92.77%)	
Yes	34 (6.83%)	22 (6.63%)	12 (7.23%)	
Brain+Bone+Liver+Lung				0.219
No	495 (99.40%)	329 (99.10%)	166 (100.00%)	
Yes	3 (0.60%)	3 (0.90%)	0 (0.00%)	
Distant metastasis				0.651
No	114 (22.892%)	78 (23.494%)	36 (21.687%)	
Yes	384 (77.108%)	254 (76.506%)	130 (78.313%)	
Sum of distant organ metastasis				0.312
Blank	69 (13.86%)	42 (12.65%)	27 (16.27%)	
1 Metastatic site	275 (55.22%)	185 (55.72%)	90 (54.22%)	
2 Metastatic sites	113 (22.69%)	80 (24.10%)	33 (19.88%)	
3 Metastatic sites	38 (7.63%)	22 (6.63%)	16 (9.64%)	
4 Metastatic sites	3 (0.60%)	3 (0.90%)	0 (0.00%)	
Sum of distant organ metastasis (2 category)				0.631
≤2 Metastatic sites	344 (69.08%)	227 (68.37%)	117 (70.48%)	
>2 Metastatic sites	154 (30.92%)	105 (31.63%)	49 (29.52%)	

LMS, leiomyosarcoma.

### Survival Analyses for Overall Survival and Cancer-Specific Survival in the Whole Cohort

**Figure 3:**
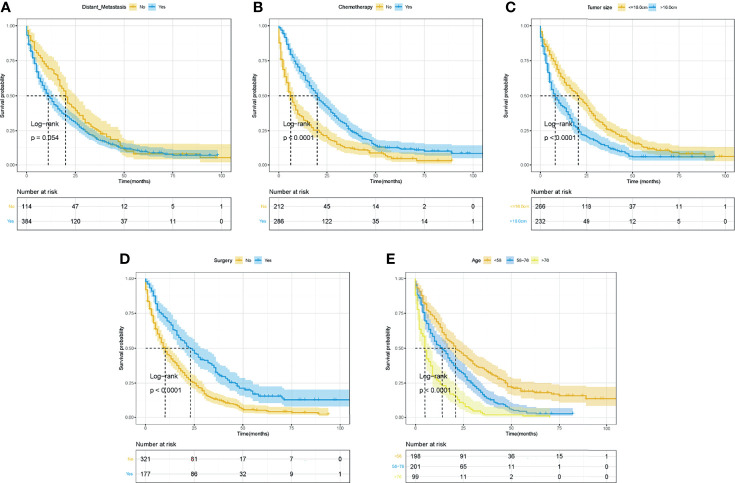
The Kaplan–Meier curves for OS based on variables for the nomograms. **(A)** Distant metastasis. **(B)** Chemotherapy. **(C)** Tumor size. **(D)** Surgery. **(E)** Age. ROC, receiver operating curve; OS, Overall Survival.

**Figure 4:**
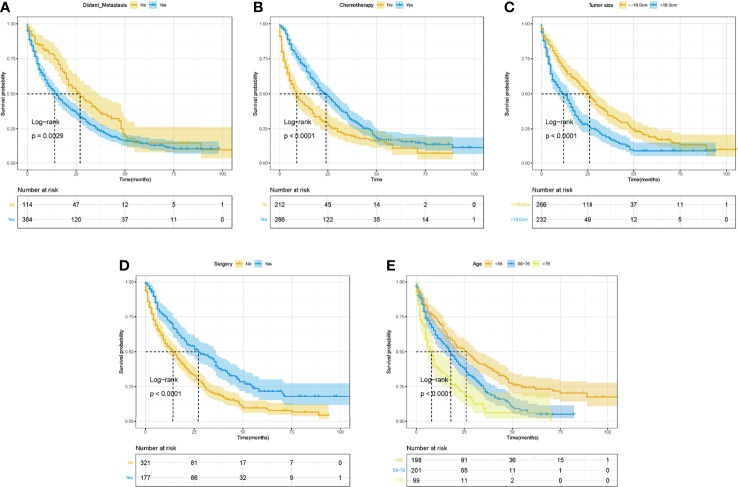
The Kaplan–Meier curves for CSS based on variables for the nomograms. **(A)** Distant metastasis. **(B)** Chemotherapy. **(C)** Tumor size. **(D)** Surgery. **(E)** Age. ROC, receiver operating curve; OS, Overall Survival.

The Kaplan–Meier curves and log-rank tests were performed to identify the possible prognostic predictors for OS and CSS. Among all variables, four variables were simultaneously observed to be associated with OS and CSS, including surgery, chemotherapy, age, and tumor size, while distant metastasis was also observed to be associated with CSS. The survival curves of OS and CSS of these five prognostic variables were shown in **Figures 3**, **4**. Except for the abovementioned five prognostic factors, other variables, including primary tumor site **(** **Supplementary Figure S1** **)**, were not significantly correlated with OS and CSS in LMS patients, including sex, year of diagnosis, race, regional lymph node surgery, radiotherapy, primary site, sum of distant organ metastasis, etc.

### Relationship Between Metastatic Sites, Number of Metastases, and Survival in Leiomyosarcoma Patients With Distant Metastasis

On the basis of the occurrence of distant metastasis, the whole cohort was divided into two subgroups, including the distant metastasis group (n = 384) and the non-distant metastasis group (n = 114). Kaplan–Meier curves and log-rank tests were conducted to explore the relationship of metastatic sites, number of metastases, and survival in LMS patients with distant organ metastasis **(** **Supplementary Figures S2**–**S5** **)**. For patients with distant organ metastasis, we found that patients who simultaneously got bone and liver metastasis or simultaneously got bone, liver, and lung metastasis were prone to get obviously shorter OS **(** **Supplementary Figure S2** **)** and CSS **(** **Supplementary Figure S4** **)** than their counterparts (all P-value <0.05). Besides this, other metastatic sites and the number of metastases did not exert obvious influence on the survival of LMS patients in this cohort.

### Relationship Between Treatment Modality and Overall Survival in Leiomyosarcoma Patients With Different Distant Organ Metastases

To determine the effective therapeutic regimens for patients who have already developed distant organ metastasis, these patients were further divided into seven subgroups based on the metastatic sites, including lung metastasis only (n = 141), bone metastasis only (n = 36), liver metastasis only (n = 98), bone+liver (n = 46), bone+lung (n = 66), liver+lung (n = 104), and bone+liver+lung (n = 34). Kaplan–Meier curves, log-rank tests, and univariate and multivariate Cox proportional hazards regression analyses were performed to determine the relationship between treatment modality and OS. The detailed information of univariate and multivariate Cox analyses for OS based on treatment modality was presented in **Supplementary Table S1**, and the survival curves of different treatment modalities in different subgroups were presented in **Supplementary Figures S6**–**S12**. Except for the subgroup of bone metastasis only (marginally significant in the subgroup of bone+liver+lung, P = 0.059), chemotherapy was an independent prognostic factor for OS in all subgroups, and patients who received chemotherapy achieved better OS than their counterparts. In contrast, radiotherapy and surgery positively associated with OS only in some subgroups. To be specific, radiotherapy was positively associated with the improvement of OS in the subgroup of liver metastasis only and liver+lung, while surgery was positively associated with the improvement of OS in the subgroup of lung metastasis only and liver metastasis only.

### Identification of Prognostic Predictors for Overall Survival and Cancer-Specific Survival in Leiomyosarcoma Patients With Metastasis

Univariate and multivariate Cox analyses were utilized to identify the potential independent prognostic predictors for OS and CSS in the training set, which are presented in **Table 3**. According to the results of univariate Cox analyses, marital status, surgery, chemotherapy, age, and tumor size were found to associate with OS. In multivariate analysis, they were further confirmed as independent prognostic factors for OS except for marital status. Furthermore, in the training set, the variables of surgery, chemotherapy, age, tumor size, and distant metastasis were identified as prognostic factors for CSS in the univariate and multivariate Cox analysis.

**Table 3:** Univariate and multivariate Cox proportional hazards regression analyses for OS and CSS in the training dataset.

Variables	Univariate analysis		Multivariate analysis			
	OS	CSS	OS		CSS	
	P-value	P-value	HR (95% CI)	P-value	HR (95% CI)	P-value
Sex (Women vs. Men)	0.872	0.943				
Year of diagnosis (2013–2015 vs. 2010–2012)	0.964	0.528				
Race						
Black vs. White	0.767	0.516				
Other vs. White	0.081	0.115				
Marital status						
Not Married vs. Married	0.657	0.966	1.153 (0.847–1.569)	0.367	1.051 (0.744–1.486)	0.776
Other vs. Married	0.015	0.024	1.058 (0.806–1.389)	0.684	1.136 (0.841–1.535)	0.405
Regional lymph node surgery (Yes vs. No)	0.217	0.315				
Surgery (Yes vs. No)	<0.01	<0.01	0.563 (0.414–0.766)	<0.01	0.629 (0.444–0.892)	0.009
Radiotherapy (Yes vs. No)	0.184	0.219				
Chemotherapy (Yes vs. No)	<0.01	0.001	0.563 (0.419–0.756)	<0.01	0.622 (0.447–0.867)	0.005
Systemic therapy (Yes vs. No)	<0.01	<0.01	0.947 (0.652–1.376)	0.776	0.982 (0.652–1.479)	0.931
AJCC T						
T1 vs. T0	0.440	0.432				
T2 vs. T0	0.594	0.436				
Tx vs. T0	0.834	0.617				
AJCC N						
N1 vs. N0	0.324	0.727				
N2 vs. N0	0.075	0.142				
Primary site						
Retroperitoneum+Peritoneum vs. Extremity+Trunk	0.508	0.096				
Others vs. Extremity+Trunk	0.296	0.152				
Age						
58–76 vs. <58	<0.01	0.003	1.733 (1.325–2.266)	<0.01	1.543 (1.153–2.065)	0.004
>76 vs. <58	<0.01	<0.01	2.739 (1.917–3.913)	<0.01	2.453 (1.642–3.665)	<0.01
Tumor size (>16.0cm vs. ≤16.0cm)	<0.01	0.01	1.791 (1.422–2.255)	<0.01	1.720 (1.320–2.242)	<0.01
Distant metastasis (Yes vs. No)	0.060	0.017			1.410 (1.021–1.949)	0.037
Sum of distant organ metastasis						
3 vs. 4	0.443	0.680				
2 vs. 4	0.472	0.741				
1 vs. 4	0.355	0.717				
Blank vs. 4	0.450	0.784				
Sum of distant organ metastasis (2 category) (>2 vs. ≤ 2)	0.420	0.658				

OS, overall survival; CSS, cancer-specific survival; AJCC, American Joint Committee on Cancer.

In summary, variables of surgery, chemotherapy, age, and tumor size were identified as independent predictors for OS and CSS, and distant metastasis was also independently associated with CSS.

### Prognostic Nomogram Establishment, Validation, and Performance Assessment

**Figure 5:**
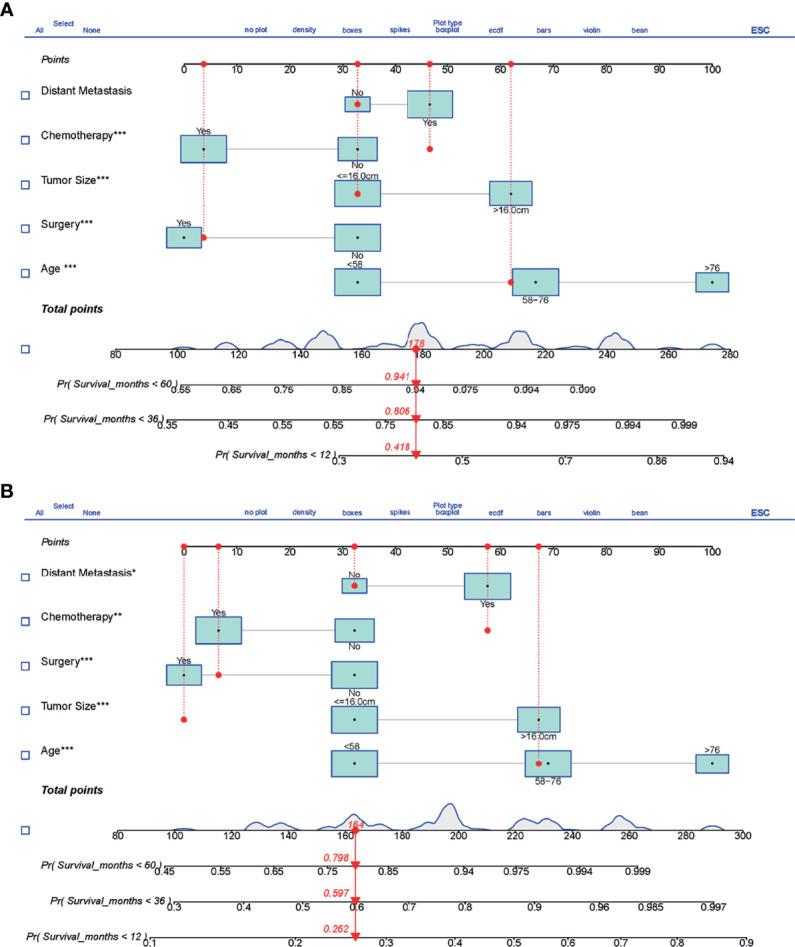
The prognostic nomograms predicting 1-, 3-, and 5-year survival for LMS patients with metastasis. **(A)** Nomogram for OS. **(B)** Nomogram for CSS. LMS, leiomyosarcoma; OS, overall survival; CSS, cancer-specific survival. The symbols *, ** and *** means the importance of this variable for the prognostic nomograms. They represent important, very important and extremely important, respectively. ROC, receiver operating curve; OS, Overall Survival.

Based on the results of univariate and multivariate Cox analyses, surgery, chemotherapy, age, tumor size, and distant metastasis were finally identified and included to establish the prognostic nomograms for OS and CSS. The visualization of the prognostic nomograms for OS and CSS was shown in **Figure 5**. With these prognostic nomograms, the 1-, 3-, and 5-year survival probability of the individual patients can be easily calculated.

**Figure 6:**
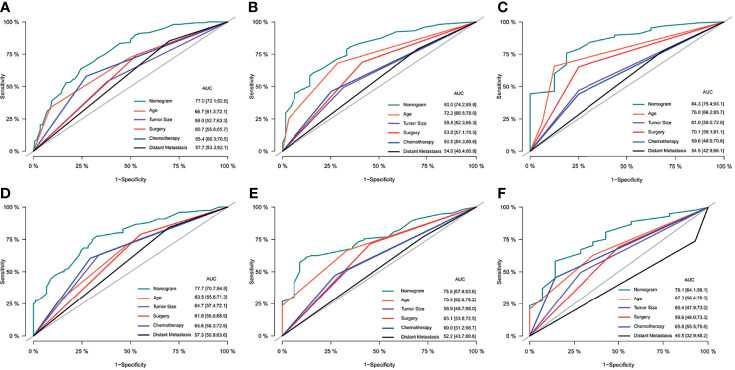
The comparison of ROC curves of the nomogram and its constituent variables (age, tumor size, surgery, chemotherapy, and distant metastasis) for predicting 1-year **(A)**, 3-year **(B)**, and 5-year **(C)** OS in the training set. The comparison of ROC curves of the nomogram and its constituent variables (age, tumor size, surgery, chemotherapy, and distant metastasis) for predicting 1-year **(D)**, 3-year **(E)**, and 5-year **(F)** OS in the validation set. ROC, receiver operating curve; OS, Overall Survival.

**Figure 7:**
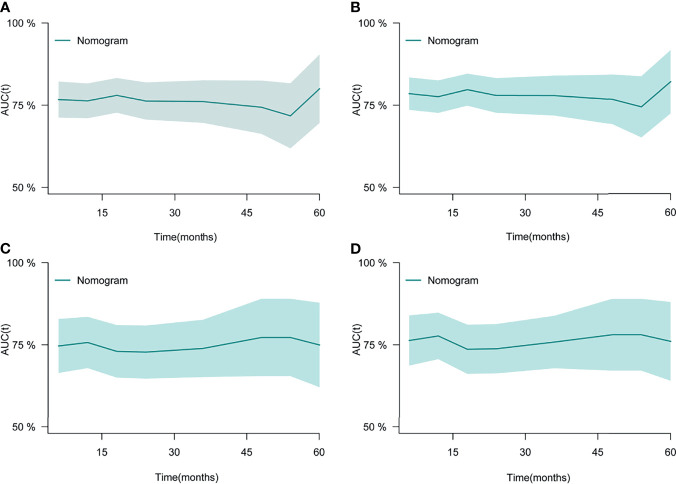
The time-dependent ROC curves of the nomogram for OS in the training set **(A)** and validation set **(C)**. The time-dependent ROC curves of the nomogram for CSS in the training set **(B)** and validation set **(D)**. ROC, receiver operating curve; OS, Overall Survival.

**Figure 8:**
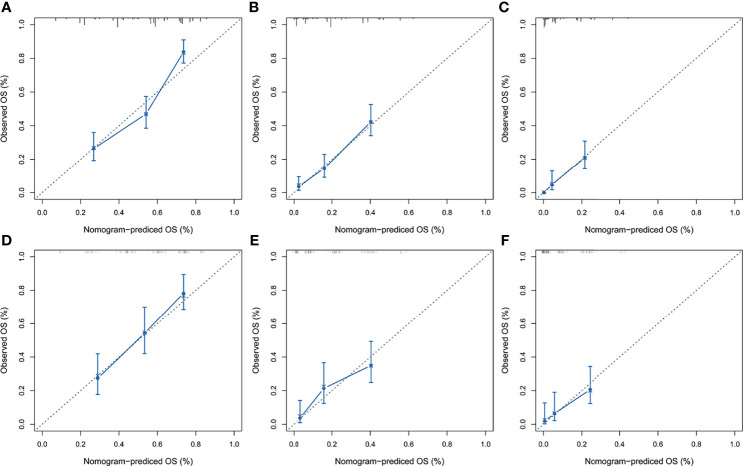
The calibration curves of the prognostic nomogram for predicting 1-year **(A)**, 3-year **(B)**, and 5-year **(C)** OS in the training set. Calibration curves of the prognostic nomogram for predicting 1-year **(D)**, 3-year **(E)**, and 5-year **(F)** OS in the validation set. ROC, receiver operating curve; OS, overall survival.

**Figure 9:**
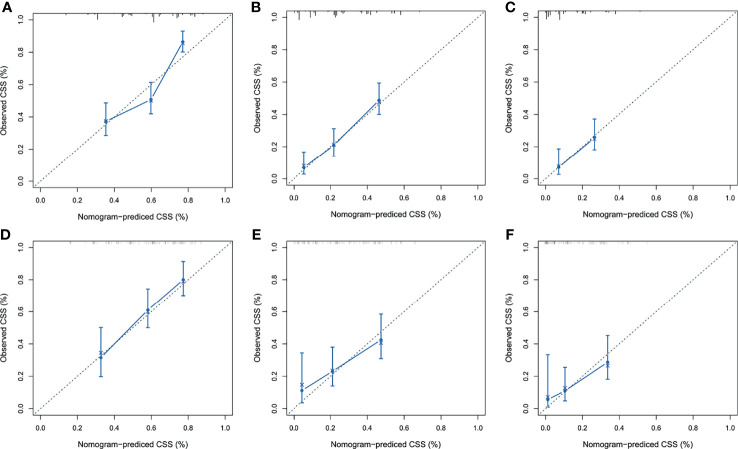
The calibration curves of the prognostic nomogram for predicting 1-year **(A)**, 3-year **(B)**, and 5-year **(C)** CSS in the training set. Calibration curves of the prognostic nomogram for predicting 1-year **(D)**, 3-year **(E)**, and 5-year **(F)** CSS in the validation set. CSS, cancer-specific survival. ROC, receiver operating curve; OS, Overall Survival.

**Figure 10:**
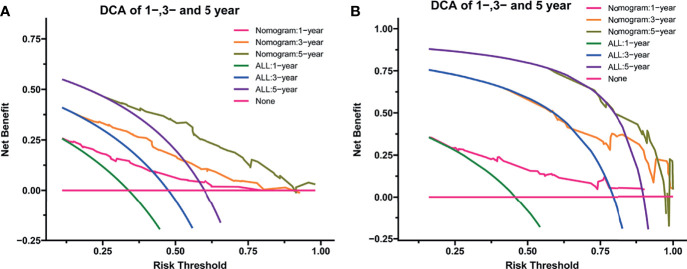
The decision curve analysis of the nomogram for predicting 1-year, 3-year, and 5-year OS in the training set **(A)** and validation set **(B)**. OS, overall survival.

After the establishment of prognostic nomograms for OS and CSS, a series of methods were utilized to validate and assess the predictive performance of these nomograms, including the ROC curves, time-dependent AUCs, calibration curves, and DCA curves. The ROC curves were firstly used to evaluate the discriminability of prognostic nomograms, and the AUCs of the nomogram for predicting 1-, 3-, and 5-year OS were 0.770, 0.800, and 0.843, respectively. The ROC curves of the nomogram and its constituent variables for OS were shown in **Figures 6A–C**. Similarly, the AUCs of the nomogram for predicting 1-, 3-, and 5-year CSS were 0.777, 0.758, and 0.761, respectively. The ROC curves of the nomogram and its constituent variables for CSS were shown in **Figures 6D–F**. The AUCs of nomograms for OS and CSS were all better than those of the constituent variables of nomograms in predicting 1-, 3-, and 5-year survival. The time-dependent AUCs also showed that the AUCs for OS and CSS in the training set and validation set were all over 0.75, indicating the favorable and acceptable discriminative power of nomograms. The time-dependent AUCs for OS and CSS in the training set and validation set were shown in **Figure 7**. The calibration curves of the nomograms for OS **(** **Figure 8** **)** and CSS **(** **Figure 9** **)** in the training set and validation set all showed excellent consistency between observed survival and nomogram-predicted survival. The DCA curves of the nomogram for 1-, 3-, and 5-year OS have exhibited a wide range of threshold probabilities for net benefit **(** **Figure 10** **)**, indicating the favorable predicting performance of prognostic nomograms for metastatic LMS patients.

### Kaplan–Meier Survival Analyses Based on Risk Stratification From Prognostic Nomograms

**Figure 11:**
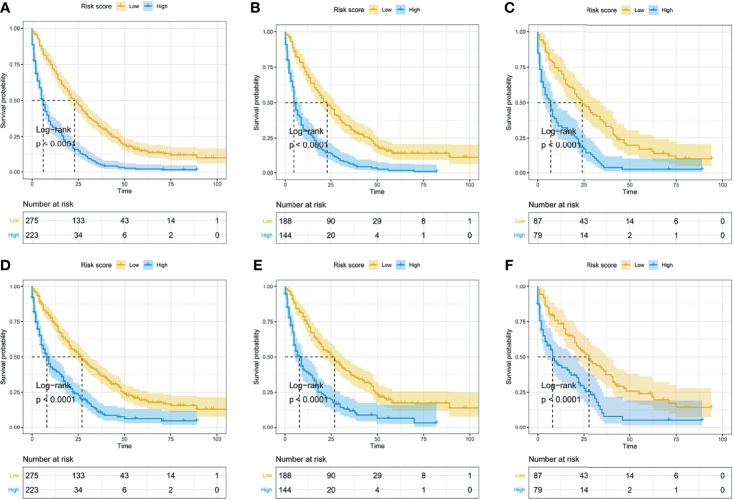
The Kaplan–Meier curves for OS and CSS based on risk score grouping from the nomogram. According to the median of risk scores in the entire cohort, the whole cohort was divided into two subgroups (low-risk group and high-risk group). The Kaplan–Meier curves for OS **(A)** and CSS **(D)** in the whole cohort. The Kaplan–Meier curves for OS **(B)** and CSS **(E)** in the training set. The Kaplan–Meier curves for OS **(C)** and CSS **(F)** in the validation set.

Based on the median of risk scores of all LMS patients, the entire cohort, training cohort, and validation cohort were divided into two subgroups, namely, the low-risk group and high-risk group. Kaplan–Meier curves and log-rank test were performed to further validate the prognostic value of the established nomograms. The survival curves for OS and CSS based on risk score grouping were presented in **Figure 11**. Significant differences in OS and CSS were observed between the two subgroups in the entire cohort, the training cohort, and the validation cohort (all P-value <0.05), suggesting the favorable survival-predicting performance of the two nomograms.

## Discussion

LMS is an aggressive and malignant type of soft tissue sarcoma originating from smooth muscle cells (1, 19). One of the most outstanding characteristics of LMS is the development of metastasis, including local metastasis and distant metastasis. Previous studies have indicated that almost 30% of LMS patients are prone to develop distant organ metastasis, among which the lung is the most frequently involved metastatic organ (12, 20, 21). Not only in LMS, but also in other malignant tumors, once patients develop metastasis, especially distant organ metastasis, their survival will reduce drastically compared with their counterparts without distant organ metastasis (8). Therefore, metastasis is deemed as one of the most paramount prognosticators for LMS, and it is included in almost all prognostic models in previous studies for initially diagnosed LMS patients. In the present study, 384 (77.11%) LMS patients got distant organ metastasis, and they finally harvested obviously worse prognosis than that of the 114 (22.89%) LMS patients who only got local metastasis. For LMS patients with local metastatic lesions, surgical resection followed by radiotherapy, chemotherapy, and sometimes in combination with targeted therapy can effectively prolong and improve the survival of these patients. However, for patients who have already suffered distant metastasis, the scene of their outcomes still remains unknown. It is reasonable to speculate empirically that patients with more distant organ metastatic sites are prone to get worse prognosis than patients with less. Interestingly, as opposed to what had been expected initially, once patients have developed distant organ metastasis, there existed no differences in OS and CSS in patients with only one organ metastasis and patients with more than one organ metastatic lesion. Both in the whole cohort and in the cohort of distant metastasis, the entire cohort can be divided into five subgroups or two subgroups on the basis of the number of metastatic sites, but no significant differences in survival were found in these subgroups. These facts indicated that the occurrence of distant metastasis, rather than the number of distant metastases, was significantly associated with the prognosis of LMS patients. In summary, two preliminary speculations can be obtained from the abovementioned facts. One is that distant metastasis is quite a pivotal prognosticator for the survival of LMS patients, and patients with distant organ metastasis get relatively shorter survival time. The other one is that, for patients who have already suffered distant organ metastasis, the survival of those with only one distant metastatic site was almost equivalent to those with more than one distant metastatic site. Several reasons may account for this phenomenon. First, curative surgery can also be performed in some of LMS patients with localized metastasis sometimes while the patients with distant organ metastasis usually have lost the opportunity to receive curative surgery, which finally leads to the reduction of survival in these patients. Second, patients with distant metastasis usually develop more severe debilitating muscle-wasting syndrome (also known as cachexia) that significantly weakens tolerance to antineoplastic therapy, results in poor prognosis, and accelerates death while with no effective treatments (22). Finally, distant metastasis is usually accompanied by chemoresistance that leads to decreased therapeutic effects under the same dosage of antitumor drugs. For those metastatic cells that have successfully colonized in other tissues, they have obtained high vitality and strong resistance due to the tremendous stresses provided by different internal environments, such as antitumor immunity, reactive oxygen species, and inflammatory responses (23). The enhanced vitality and resistance of metastatic cells are responsible for increased chemoresistance and decreased antineoplastic effects, finally leading to the deterioration of prognosis.

It is still a huge challenge for oncologists to seek effective treatment modalities for patients who have already developed distant metastasis. In our study, based on the results from Kaplan–Meier curves, log-rank test, and Cox regression analysis, we found that only chemotherapy can definitely improve the prognosis of LMS patients in all subgroups, which was consistent with the results of previous studies (24). Besides, we also found that radiotherapy was positively associated with the improvement of OS in the subgroup of liver metastasis only and liver+lung, and surgery was positively associated with the improvement of OS in the subgroup of lung metastasis only and liver metastasis only. Similar to patients in other study (13), only patients with solitary metastasis in our study may own the opportunity to receive surgery and may get improved OS. In other words, in this study, the surgery for LMS patients may be symptom-improving, rather than survival-improving, in patients who have already suffered from metastasis. For patients with multiple distant organ metastases, chemotherapy plus radiotherapy may be an effective method to alleviate symptoms and improve survival, but further validation is needed in future studies.

In this study, based on the results of univariate and multivariate Cox regression analysis, five prognostic factors were finally identified to be independently associated with OS and CSS in LMS patients, including distant metastasis, surgery, chemotherapy, tumor size, and age. Previous studies had indicated that an increase of age was closely associated with a decrease in the survival of LMS patients (25, 26). In the present study, we also found a similar phenomenon that patients with age over 58 years and 76 years got obviously worse OS and CSS rates. A more advanced age usually forebodes the degeneration and senescence of major organs of the human body, especially the immune system, which is responsible for restricting and eliminating the tumor cells in the human body (27). In older LMS patients, a weakened or impaired immune system may largely increase the opportunity for tumor cells to transfer to other sites to form a new metastatic niche, finally leading to the multiple colonization of cancer cells in other tissues. Consistent with previous studies, our study also suggested that larger tumor size (>16.0 cm) was related to poorer prognosis in LMS patients. In clinical practice, larger tumors often indicate more difficulties to remove tumor completely and get R0 resection margins. Meanwhile, larger tumors are usually accompanied by abundant neovascularization, which tremendously increases the risks of hematogenous metastasis because of the extrusion during surgical procedures. With regard to the therapeutic regimens of LMS, surgery and chemotherapy remain the most paramount local and systemic treatments for LMS patients. For patients without distant organ metastasis and patients with solitary metastasis, surgical resection combined with the succeeding systemic therapy is the optimal therapeutic strategy to obtain longer survival. Qian et al. (24) have concluded in their study that surgery combined with chemotherapy can improve the survival of patients with extremity LMS and metastasis at initial diagnosis. Except for the purpose of obtaining longer survival, surgical resection is also crucial for patients with unresectable metastatic lesions, as cytoreductive surgery can get other treatment effects, such as alleviating the symptom of pain and improving the quality of life (28, 29). Therefore, surgical resection should be emphasized in patients with resectable metastases and in patients with severe symptoms. Chemotherapy is also a major constituent in multidisciplinary treatment strategies, but its effects are highly associated with the sensitivities to chemotherapeutic agents in tumor cells and the tolerance of chemotherapy-related toxicities in human bodies. Some previous studies have indicated that chemotherapy may not benefit older soft tissue sarcoma patients in relapse-free survival or overall survival (30), but in a relatively younger cohort, patients who received surgery plus adjuvant chemotherapy can harvest significantly prolonged survival than those who were treated with surgery alone (31, 32). Similarly, in this study, chemotherapy was proven to be a significant predictor and resulted in better prognosis, indicating that chemotherapy is beneficial in prolonging the survival of LMS patients with metastasis.

In this study, five prognostic predictors were finally confirmed as independent predictors for OS and CSS, and they were further included to construct the prognostic nomograms for survival. The performance of the prognostic nomograms was evaluated by the methods of ROC, time-dependent ROC curves, calibration curve, and DCA curve. In the evaluation of discrimination ability, the AUCs of the nomogram for predicting 1-, 3-, and 5-year OS and CSS were significantly higher than the AUCs of its constituent variables. Similarly, the AUCs of the nomograms were also over 0.75 when dynamically predicting the OS and CSS both in the training set and in the validation set, suggesting the favorable discriminatory ability of the established nomograms in our study. Although previous research has reported several prediction nomograms for LMS patients, such as extremity LMS (7) and uterine LMS (25), these studies mainly focused on one subset of LMS. Our study was the first one to investigate the prognostic predictors and construct prediction models in a cohort of LMS patients with metastasis. Compared with the nomograms reported in previous studies, the prognostic predictors included in the nomograms were different from those of other studies (12, 33). The reason for this is that our nomograms are based on a cohort of metastatic LMS patients, and thus some vital prognosticators reported in previous studies, such as race, primary site, grade, and T stage, were no longer statistically significant prognosticators in this study (12). Even so, our nomograms also got a favorable and sufficient discriminatory power when compared with the existing nomograms, which can enable oncologists to assess individualized survival probability and help to optimize therapeutic strategies. With regard to the accuracy of prediction models, the nomogram-predicted 1-, 3-, and 5-year OS and CSS probability was highly consistent with the actual observed 1-, 3-, and 5-year OS and CSS probability, which indicated that the established nomogram in this study was highly reliable and accurate. In addition, the DCA curves of the nomogram in predicting survival obtained a relatively wide range of net benefit, demonstrating that more LMS patients may benefit from the nomogram-based clinical intervention in clinical practice. Furthermore, we calculated the risk scores of survival for all LMS patients based on the established prediction nomogram. In our study, the entire cohort was divided into low-risk group and high-risk group based on the median of risk scores of all LMS patients. The two subgroups got obviously different survival times whether in the entire cohort or in the training set or validation set, which further provided additional evidence supports for the favorable prediction performances of the established nomogram in this study. In summary, we established prognostic nomograms by incorporating five independent predictors in this study, and the nomograms were proven to be of sufficient discriminatory power and high accuracy and reliability, as well as to have a wide range of net benefit threshold, which can facilitate oncologists to make accurate risk stratification and help to optimize prognosis-based decision-making in clinical practice.

Similar to other studies based on SEER database, our study inevitably contains some limitations. Firstly, in order to ensure the completeness of the variables included in this study, strict inclusive and exclusive criteria were adopted for LMS patients, which may lead to some statistical bias for the characteristics of a retrospective study. Secondly, some other pivotal variables, such as serum biochemical indicators, immunoregulatory indices, and peripheral blood lymphocyte subsets, cannot be extracted from the SEER database, and therefore these variables were not included in the established nomograms, which may to some extent restrict the further improvement of the prognostic nomograms. Finally, although the established nomograms in this study presented favorable accuracy and reliability in the training set and validation set, they were built based on a public database, and thus further validation of the prognostic models is necessary with the real-world dataset from other research institutes.

## Conclusions

No significant differences in OS were observed in patients with distant organ metastasis and in patients with localized metastasis. For patients who have already developed distant organ metastasis, the sites and number of metastases seem to be not closely associated with survival. Chemotherapy seems to be an effective treatment in prolonging survival in all metastatic LMS patients. In contrast, only a specific proportion of metastatic LMS patients can benefit from radiotherapy and surgery. Five prognostic factors were finally identified to be independently associated with the survival of LMS patients, and they were further included to establish prognostic nomograms. The established nomograms have presented excellent discriminability and high accuracy and consistency, which can serve as an effective and reliable assessment tool for oncologists to perform risk stratification and optimize treatment options. To generalize the utilization of prognostic nomograms, further validation is still warranted.

## Data Availability Statement

The dataset used to perform the statistical analysis is available in the SEER database (https://seer.cancer.gov/).

## Author Contributions

QY and YZ conceived and designed the study and completed the article. QY, YZ, YW, and ZY performed the statistical analysis and explained the results in this study. CZ and HA were responsible for the figures and tables in this study. FL scrutinized the whole process of this study and critically reviewed the initial article. All authors contributed to the article and approved the submitted version.

## Funding

This study was supported by the talent funds of Southwest Hospital (No. XZ-2019-505-021).

## Conflict of Interest

The authors declare that the research was conducted in the absence of any commercial or financial relationships that could be construed as a potential conflict of interest.

## Publisher’s Note

All claims expressed in this article are solely those of the authors and do not necessarily represent those of their affiliated organizations, or those of the publisher, the editors and the reviewers. Any product that may be evaluated in this article, or claim that may be made by its manufacturer, is not guaranteed or endorsed by the publisher.

## Abbreviations

LMS, leiomyosarcoma; OS, overall survival; CSS, cancer-specific survival; SEER, Surveillance, Epidemiology, and End Results; ROC, receiver operating characteristic; DCA, decision curve analysis.
