# Supplementary material for: Prognosticators and Prognostic Nomograms for Leiomyosarcoma Patients With Metastasis
Source: Front Oncol. 2022 Mar 18;12:840962. doi: 10.3389/fonc.2022.840962 (PMC8971727; doi:10.3389/fonc.2022.840962)
Supplement: Supplementary file 1 [file DataSheet_1.doc]

**Supplementary Materials**

**S.Table 1.** Univariate and multivariate Cox proportional hazards regression analyses for OS of LMS patients in subgroups with distant organ metastasis

**S.Figure 1.** The Kaplan-Meier curves for OS of LMS patients based on primary tumor site.

**S.Figure 2.** The Kaplan-Meier curves for OS of LMS patients in the subgroup of distant organ metastasis. Influences of sites and number of metastasis on the survival of LMS patients. (A)bone metastasis only; (B)bone+liver; (C)bone+liver+lung; (D)bone+lung; (E)brain+bone; (F)brain+bone+liver; (G)brain+bone+liver+lung; (H)brain+bone+lung; (I)brain+liver.

**S.Figure 3.** The Kaplan-Meier curves for OS of LMS patients in the subgroup of distant organ metastasis. Influences of sites and number of metastasis on the survival of LMS patients. (A)brain+lung; (B)liver metastasis only; (C)liver+lung; (D)lung metastasis only; (E)sum of distant organ metastasis(two category); (F)sum of distant organ metastasis.

**S.Figure 4.** The Kaplan-Meier curves for CSS of LMS patients in the subgroup of distant organ metastasis. Influences of sites and number of metastasis on the survival of LMS patients. (A)bone metastasis only; (B)bone+liver; (C)bone+liver+lung; (D)bone+lung; (E)brain+bone; (F)brain+bone+liver; (G)brain+bone+liver+lung; (H)brain+bone+lung; (I)brain+liver.

**S.Figure 5.** The Kaplan-Meier curves for CSS of LMS patients in the subgroup of distant organ metastasis. Influences of sites and number of metastasis on the survival of LMS patients. (A)brain+lung; (B)liver metastasis only; (C)liver+lung; (D)lung metastasis only; (E)sum of distant organ metastasis(two category); (F)sum of distant organ metastasis.

**S.Figure 6.** The Kaplan-Meier curves for OS of LMS patients in the subgroup with only lung metastasis. (A)Surgery; (B)Radiotherapy; (C)Chemotherapy.

**S.Figure 7.** The Kaplan-Meier curves for OS of LMS patients in the subgroup with only bone metastasis. (A)Surgery; (B)Radiotherapy; (C)Chemotherapy.

**S.Figure 8.** The Kaplan-Meier curves for OS of LMS patients with only liver metastasis. (A)Surgery; (B)Radiotherapy; (C)Chemotherapy.

**S.Figure 9.** The Kaplan-Meier curves for OS of LMS patients with bone and liver metastasis. (A)Surgery; (B)Radiotherapy; (C)Chemotherapy.

**S.Figure 10.** The Kaplan-Meier curves for OS of LMS patients with bone and lung metastasis. (A)Surgery; (B)Radiotherapy; (C)Chemotherapy.

**S.Figure 11.** The Kaplan-Meier curves for OS of LMS patients with liver and lung metastasis. (A)Surgery; (B)Radiotherapy; (C)Chemotherapy.

**S.Figure 12.** The Kaplan-Meier curves for OS of LMS patients with bone, liver, and lung metastasis. (A)Surgery; (B)Radiotherapy; (C)Chemotherapy.

Supplementary Table 1. Univariate and multivariate Cox proportional hazards regression analyses for OS in subgroups with distant organ metastasis

| Subgroups | Variables | level | N (%) | Univariate HR(95% CI) | P-value | Multivariate HR(95% CI) | P-value |
| --- | --- | --- | --- | --- | --- | --- | --- |
| Lung metastasis only (n=141) |  |  |  |  |  |  |  |
|  | Surgery | No | 82 (58.2%) | Reference |  | Reference |  |
|  |  | Yes | 59 (41.8%) | 0.426(0.294-0.618) | P<0.01 | 0.412(0.284-0.599) | P<0.01 |
|  | Radiotherapy | No | 105 (74.5%) | Reference |  |  |  |
|  |  | Yes | 36 (25.5%) | 0.795(0.529-1.195) | 0.270 |  |  |
|  | Chemotherapy | No | 61 (43.3%) | Reference |  | Reference |  |
|  |  | Yes | 80 (56.7%) | 0.490(0.342-0.701) | P<0.01 | 0.471(0.329-0.674) | P<0.01 |
| Bone metastasis only (n=36) |  |  |  |  |  |  |  |
|  | Surgery | No | 17 (47.2%) | Reference |  |  |  |
|  |  | Yes | 19 (52.8%) | 0.744(0.374-1.478) | 0.398 |  |  |
|  | Radiotherapy | No | 15 (41.7%) | Reference |  |  |  |
|  |  | Yes | 21 (58.3%) | 1.690(0.796-3.588) | 0.172 |  |  |
|  | Chemotherapy | No | 19 (52.8%) | Reference |  |  |  |
|  |  | Yes | 17 (47.2%) | 0.754(0.379-1.498) | 0.420 |  |  |
| Liver metastasis only (n=98) |  |  |  |  |  |  |  |
|  | Surgery | No | 70 (71.4%) | Reference |  | Reference |  |
|  |  | Yes | 28 (28.6%) | 0.393(0.238-0.650) | P<0.01 | 0.389(0.234-0.645) | P<0.01 |
|  | Radiotherapy | No | 84 (85.7%) | Reference |  | Reference |  |
|  |  | Yes | 14 (14.3%) | 0.429(0.227-0.813) | 0.009 | 0.456(0.240-0.867) | 0.017 |
|  | Chemotherapy | No | 39 (39.8%) | Reference |  | Reference |  |
|  |  | Yes | 59 (60.2%) | 0.631(0.412-0.966) | 0.034 | 0.559(0.364-0.859) | 0.008 |
| Bone+Liver (n=46) |  |  |  |  |  |  |  |
|  | Surgery | No | 41 (89.1%) | Reference |  |  |  |
|  |  | Yes | 5 (10.9%) | 0.827(0.325-2.107) | 0.691 |  |  |
|  | Radiotherapy | No | 28 (60.9%) | Reference |  | Reference |  |
|  |  | Yes | 18 (39.1%) | 2.203(1.154-4.207) | 0.017 | 1.673(0.855-3.275) | 0.133 |
|  | Chemotherapy | No | 22 (47.8%) | Reference |  | Reference |  |
|  |  | Yes | 24 (52.2%) | 0.350(0.187-0.656) | 0.001 | 0.404(0.209-0.780) | 0.007 |
| Bone+Lung (n=66) |  |  |  |  |  |  |  |
|  | Surgery | No | 52 (78.8%) | Reference |  |  |  |
|  |  | Yes | 14 (21.2%) | 0.676(0.363-1.257) | 0.216 |  |  |
|  | Radiotherapy | No | 41 (62.1%) | Reference |  |  |  |
|  |  | Yes | 25 (37.9%) | 1.262(0.751-2.121) | 0.379 |  |  |
|  | Chemotherapy | No | 26 (39.4%) | Reference |  | Reference |  |
|  |  | Yes | 40 (60.6%) | 0.293(0.168-0.512) | P<0.01 | 0.293(0.168-0.512) | P<0.01 |
| Liver+lung (n=104) |  |  |  |  |  |  |  |
|  | Surgery | No | 83 (79.8%) | Reference |  |  |  |
|  |  | Yes | 21 (20.2%) | 0.815(0.497-1.336) | 0.418 |  |  |
|  | Radiotherapy | No | 83 (79.8%) | Reference |  | Reference |  |
|  |  | Yes | 21 (20.2%) | 2.012(1.224-3.310) | 0.006 | 1.973(1.197-3.252) | 0.008 |
|  | Chemotherapy | No | 33 (31.7%) | Reference |  | Reference |  |
|  |  | Yes | 71 (68.3%) | 0.615(0.399-0.947) | 0.027 | 0.628(0.408-0.968) | 0.035 |
| Bone+Liver+lung (n=34) |  |  |  |  |  |  |  |
|  | Surgery | No | 30 (88.2%) | Reference |  |  |  |
|  |  | Yes | 4 (11.8%) | 0.696(0.242-1.998) | 0.500 |  |  |
|  | Radiotherapy | No | 20 (58.8%) | Reference |  | Reference |  |
|  |  | Yes | 14 (41.2%) | 2.452(1.131-5.314) | 0.023 | 1.459(0.575-3.701) |  |
|  | Chemotherapy | No | 15 (44.1%) | Reference |  | Reference | 0.426 |
|  |  | Yes | 19 (55.9%) | 0.328(0.153-0.706) | 0.004 | 0.408(0.160-1.036) | 0.059 |

Abbreviations: OS, overall survival; LMS, leiomyosarcoma.
